# A low‐cost, computer‐controlled robotic flower system for behavioral experiments

**DOI:** 10.1002/ece3.2062

**Published:** 2016-03-16

**Authors:** Erno Kuusela, Juho Lämsä

**Affiliations:** ^1^ Department of EcologyUniversity of OuluPO Box 3000Oulu90014Finland

**Keywords:** Arduino, automatization, bumblebee, data acquisition, electronics, feeder, infrared sensor, microcontroller, pollination, servo

## Abstract

Human observations during behavioral studies are expensive, time‐consuming, and error prone. For this reason, automatization of experiments is highly desirable, as it reduces the risk of human errors and workload. The robotic system we developed is simple and cheap to build and handles feeding and data collection automatically. The system was built using mostly off‐the‐shelf components and has a novel feeding mechanism that uses servos to perform refill operations. We used the robotic system in two separate behavioral studies with bumblebees (*Bombus terrestris*): The system was used both for training of the bees and for the experimental data collection. The robotic system was reliable, with no flight in our studies failing due to a technical malfunction. The data recorded were easy to apply for further analysis. The software and the hardware design are open source. The development of cheap open‐source prototyping platforms during the recent years has opened up many possibilities in designing of experiments. Automatization not only reduces workload, but also potentially allows experimental designs never done before, such as dynamic experiments, where the system responds to, for example, learning of the animal. We present a complete system with hardware and software, and it can be used as such in various experiments requiring feeders and collection of visitation data. Use of the system is not limited to any particular experimental setup or even species.

## Introduction

Use of artificial flowers in pollination experiments has many advantages compared with field studies, including a comprehensive control of the environment. The easiest way of conducting such a laboratory experiment is to establish a group of artificial flowers in an experiment arena and refilling their nectar reservoirs by hand, and then making observations visually (e.g., Cartar and Real 1997; Lynn et al. 2005). However, such a system is labor‐intensive and the experimenter is required to watch the animals constantly. In comparison, the benefits of an automated system are obvious: There is no need to refill nectar manually, and data collection about visitations to flowers is more reliable using sensors rather than visual observations by the researcher. This is especially the case when dealing with rapidly moving animals, such as most pollinators.

According to Essenberg (2015), the first automatic electronic flowers were developed in the 1970s (Grossmann 1973; Hartling and Plowright 1979). Subsequently, various systems have been developed to provide sugar solution either in continuous fashion (e.g., Leadbeater and Chittka 2008; Ohashi et al. 2010) or in discrete doses, such as designs using an electromagnet (e.g., Keasar 2000; Cnaani et al. 2006; Lihoreau et al. 2010). Regardless of the type of feeding mechanism, light sensors are commonly used to track the movements of pollinators (e.g., Keasar 2000; Winter and Stich 2005; Cnaani et al. 2006; Sokolowski and Abramson 2010).

Here, we describe an artificial flower system that is fully automatic in terms of its refill operation and data collection and that uses a novel feeding mechanism to deliver discrete doses of sugar solution. The system we designed is highly reliable, relatively easy and cheap to construct, and uses mostly off‐the‐shelf components. Although the original system was developed for bumblebees (*Bombus terrestris*, L.), a common model species in behavioral and pollination experiments, its hardware parts can be scaled up or down to suit other species.

## System Description

**Figure 1:**
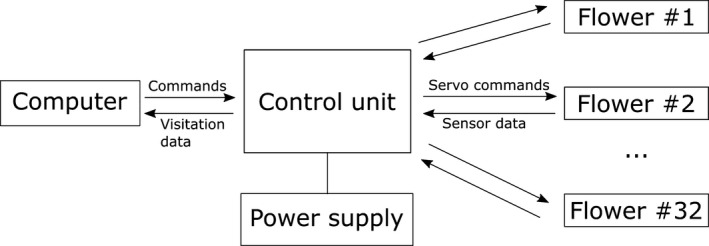
Block diagram of the system. Arrows represent data flows.

**Figure 2:**
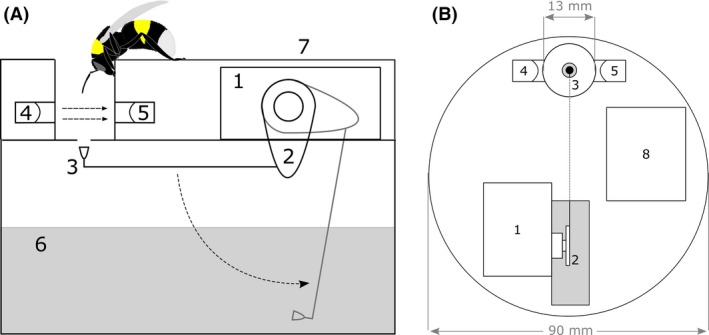
(A) Schematic side view of a single flower. (B) Schematic top view of a single flower, with top cover removed. 1. Servo. 2. Servo arm. 3. Nectar cup (in the feeding position, movement range represented by the curved dashed arrow). 4. IR LED (light path represented by two dashed arrows). 5. IR phototransistor. 6. Nectar container (left picture only). 7. Top cover (left picture only). 8. Circuit board (right picture only). The position of phototransistor and LED is correct in the right picture. In the left picture, they are positioned for illustration purposes. IR, infrared.

**Figure 3:**
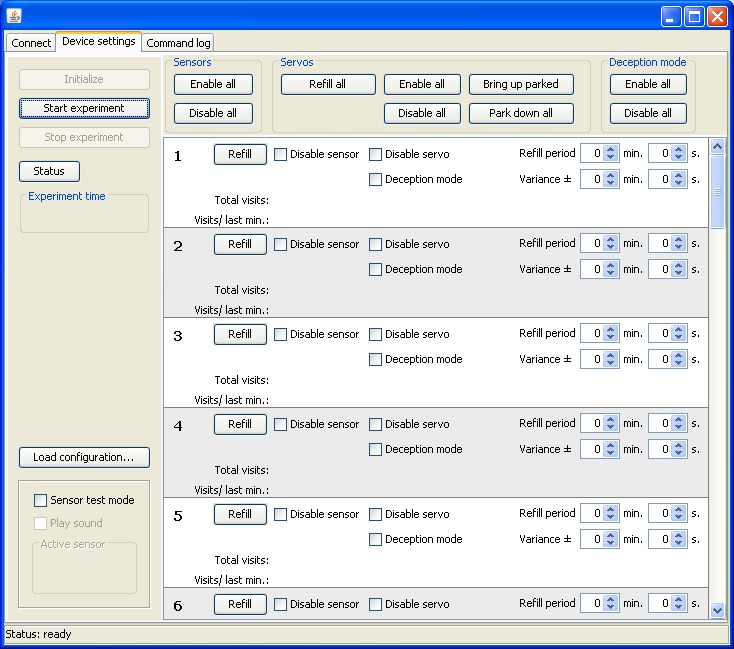
User interface of the control software.

The system includes a control unit, separate flowers, and a personal computer (Fig. 1). The function of the control unit is to handle the electronics of the flowers, collect data from them, and to send data to the computer. The flowers themselves contain an infrared (IR) sensor, their associated electronics, and an electromechanical device (servo) that offers a small, precise amount of sugar solution (referred from hereafter as “nectar”) from a reservoir (Fig. 2). The computer runs software that controls the refilling rate and data collection via the control unit (Fig. 3).

### Control unit

The main responsibilities of the control unit are as follows: (1) low‐level handling of the electronics of each flower, (2) collection of visitation data from the sensors, and (3) interpretation of the commands from the computer.

The heart of the control unit is Arduino Mega 2560 single‐board microcontroller platform (http://www.arduino.cc), which is based on ATmega2560 processor chip by Atmel (http://www.atmel.com/, San Jose, CA). The microcontroller is connected by ribbon cables to two separate connector cards which contain components needed to connect the flowers. The microcontroller is powered by USB connection to the computer, whereas flowers receive electricity from a separate power supply via connector card. Arduino Mega 2560 can simultaneously use up to 32 flowers.

We used a standard 300 W PC ATX power supply to provide +5 Volt direct current for all 32 flowers in the system. Current draw of a single flower can be up to 200 milliamperes (mA) or even more, depending upon the type of servos used. As the microcontroller platform is powered by computer's USB port, its maximum current output is limited to 500 mA, which is not enough for all flowers, and therefore, a separate power supply unit is needed.

The program running on the microcontroller (hereafter referred to as the “firmware”) handles the electronics in the system: It reads infrared sensors at specific intervals and generates correct electrical pulses to drive the servomotors. The firmware is operated by sending specific commands to it via the USB cable, which describe some simple operations, such as “refill flower #8”. The control unit sends back data collected from the sensors.

Infrared sensors are constantly read at 10 Hz frequency (i.e., 10 times per second). The control unit uses the following algorithm to determine the time and length of a visit: When an animal enters a flower, and sensor detects it, system time (seconds elapsed since the start of the experiment) is recorded. Simultaneously, a timer is started; this timer value is incremented at 100 ms intervals and zeroed after it reaches the value 10, that is, timer resets once per second. Positive readings (that is, when the animal is detected) from IR sensor are counted during this 1‐sec time period, and if detection count is greater than a certain preset value (value 2 was used in our first study), a variable containing the visit length is incremented by one. Otherwise, if detection count fails to reach this preset value, visit to flower is determined to be ended. The minimum length of a visit is 1‐sec; in reality, the actual visit may be shorter than this (for example, 0.3 sec), but the algorithm rounds value up to the next full second.

This algorithm was designed because bumblebee behavior may not be consistent during every visit; instead of going straight into the flower, for example, the animal may go in and back out quickly, and then go into the flower again. Without our algorithm, the system would detect each such erratic movement as a separate visit and interpreting the data would be much more difficult.

The control unit requires a USB connection to a computer, as its behavior is determined by the commands it receives from the computer. This greatly simplifies the design of the hardware and firmware, because the user interface for the device can be a standard desktop software instead using a “stand‐alone” device with its own LCD display, command buttons, and data storage (e.g., flash memory card).

### The flowers

Each flower in the system is a self‐contained unit and operates under the control unit's command, independently of the other flowers. Power and data connection to a flower is provided via a four‐wire cable, which was 5–10 m long in our system. The flower itself has a nectar reservoir (approx. 300 mL volume), from which the nectar cup is refilled. In our system, the nectar cup was a small Phillips screw head, which had a volume of 1.7 *μ*L. The cup is fixed to an end of a steel wire, which in turn is attached to servomotor's shaft via a servo horn (this assembly with servo horn, steel wire, and nectar cup is referred to as “servo arm”). The empty weight of a single flower is 50 g without a cable.

When in its normal position, the arm is held horizontal to position the nectar cup directly below the feeding hole. During a refill operation, the servo arm turns 60° downwards and submerges the cup into the nectar reservoir to fill it; after this, the servo is returned to the normal position. The exact position of the nectar cup can be fine‐tuned simply by bending the steel wire arm by hand. The refill cycle takes 14 sec; while a faster rate of refill is technically possible, we found during testing that this led to a periodic formation of a small air bubble in the nectar cup we used, thereby preventing it from filling up completely.

A lid covers the reservoir, and mounted on top of the lid is the servo and a small circuit board. A rectangular hole is cut to the lid, through which the servo arm operates. Nectar is available to the animal via a 3‐mm feeding hole directly above the nectar cup. A short plastic tube is vertically attached to the lid, so that the feeding hole is at the bottom of a “pit” where animal has to crawl. Mounted on the sides of the tube are an infrared LED and a phototransistor, which detect the feeding visit. Peak spectral wavelength of the IR LED is 940 nm, and the phototransistor was selected to spectrally match the LED. When the animal crawls into the sensor tube, it blocks the passage of the IR light beam, causing the phototransistor to shut off; this in turn is sensed by the control unit.

On top of the lid is the top cover, which protects the circuit board and servo. Different kinds of symbols can be attached to the top cover to symbolize, for example, flower corolla. Another function of the top cover is to hide the moving parts of the refill mechanism (servo and servo arm) so that the animals cannot visually detect when the flower is refilling the nectar cup.

### Computer and control software

The control software running on the computer is responsible for sending commands to the control unit and receiving flower visitation data from it. The software was written in Java programming language and works stably at least on Windows XP and Windows 7 operating systems.

Before starting an experiment, the system is configured using the control software. Each flower's refill rate can be individually configured, and if needed, the refill mechanism and/or sensor of specific flowers can be disabled. The refilling rate can be fixed (with the operation performed at steady intervals) or it can include random variance (within an adjustable range). Once an experiment is started, the software controls the experimental setup automatically, refilling flowers according to the predefined configuration, and gathering sensor data from them. When the user decides to stop the experiment (via the control software), the sensor data are formatted and saved automatically as a Microsoft Excel file.

When an experiment is not running, the flowers can be commanded to refill themselves manually. The servo arms can also be stopped and held in downward position, a feature that proved useful in some maintenance operations (such as cleaning of the nectar cups).

We also included a “deception mode” feature in the control software and firmware: In this mode, the servo arm is not brought to a fully upward position, so that the nectar cup is below the feeding hole but still inaccessible to the animal. The purpose of this feature is to simulate deceptive flowers, such as some orchids, which do not produce nectar. Independent flowers can be configured to be in deception mode; they respond to refill commands such as flowers in “normal” mode, but keep the nectar cup out of the reach of the animal.

### Price

See Table 1.

**Table 1:** Rough price estimate for the parts of the robotic system (with 32 flowers, not including the computer)

Flower	
Cable (5 m)	3 US$
Servo	2 US$
Electronics	3 US$
Structural parts (reservoir, lid, etc.)	2 US$
Flower total	10 US$ (10 US$ × 32 = 320 US$)
Control unit	
Arduino Mega 2560	50 US$
Electronics	20 US$
Power supply	30 US$
Control unit total	100 US$
System total	420 US$

## Discussion

We used this robotic system in two separate behavioral studies during 2014 and 2015 (J. Lämsä, E. Kuusela, S. Juntunen, A. Jäkäläniemi, P. Watts and J. Tuomi, unpubl.). A training phase was arranged in a separate nest cage with five flowers and an experiment phase conducted in a flight arena that was connected to the nest cage via a gated tube. The flight arena had 27 flowers, and the same system operated flowers in both cages simultaneously. Bumblebees readily visited the flowers. Based on our observations, the bumblebees were unable to detect the refill operations before actually probing the feeding hole.

System maintenance consisted of periodic cleaning of the flowers and exchanging of old nectar with fresh. Some of the servos in nest cage flowers had occasional malfunctions after long continuous use (several weeks of 24 hours per day operation) and were replaced. Servos failed because their internal mechanical parts wore out, which probably could be avoided by purchasing higher quality servos. However, not a single experiment flight in either of our studies failed because of these malfunctions, so overall reliability of the system can be considered good. The servo replacement was also relatively quick and easy, requiring about ten minutes of work. This delay was avoided by building spare flowers.

The servos used in this system were “micro” size class analog servos commonly used in remote‐controlled model aircrafts. These are nowadays widely available and cheap (as low as 2 US$ per piece, Table 1). Servos and all the other electronic parts can be purchased from well‐equipped electronics stores or online. Structural parts were purchased from regular hardware stores and supermarkets.

The lack of critical values in the structure of the feeding mechanism is a strength of our design. The size and positioning of parts relative to each other can vary, and once adjusted, there is virtually no variation in the repeated movements of the servo arm. Also, unlike float‐and‐magnet type feeding mechanisms (e.g., Lihoreau et al. 2010), our design avoids the problems associated with variation of fluid level in the nectar reservoir. As the servo arm is mechanically attached to the servo, there is also no risk of misalignment of the nectar cup in relation to the feeding hole as may occur when using a float‐and‐magnet design. Note, however, that these conclusions are only based on our experience with our own prototype device that used a magnet and a float, which we discarded in favor of the design presented in this paper.

The refill mechanism also avoids overfilling, whereby excess nectar accumulates in the feeding area. Overfilling might be a risk in designs that use a pump‐type delivery mechanism (e.g., Leadbeater and Chittka 2008), and while overfilling can be avoided to some degree using sensors (e.g., a new dose is delivered only after an animal has visited the flower), overfilling can still be problematic if part of the nectar dose is left in the feeding area. Also, we decided against a pump‐type design because they require high‐precision parts (e.g., syringes, reduction gears) that can be expensive.

The visitation detector is simple and reliable, and the general design has been used in other artificial flower systems (Keasar 2000; Sokolowski and Abramson 2010). By mounting the detector on the side walls of a tube where the animal has to crawl into, the risk of getting false readings is smaller than using a detector system where sensors are placed around the feeding hole on the surface of the flower.

This system can be used without modifications for a wide range of behavioral experiments with bumblebees and with minor modifications for many other pollinators. For example, the sensor tube can be scaled down to suite smaller members of the Apidae (such as honeybees). With larger structural changes to the flowers, the system could possibly be used to feed butterflies and maybe even with hummingbirds and flower bats. With major hardware changes, the system could be used as a general feeder to many other types of animals, such as mice, dogs, and pigeons, as the only critical mechanical parts are the standardized servos, which are widely available in different sizes. The flowers in the current design are lightweight and compact (90 mm diameter), offering flexibility in experimental designs.

Flowers can be scaled down by placing the parts (circuit board, sensor tube, and servo) more tightly on the lid, and using a smaller diameter nectar container. We estimate that this can reduce the flower diameter by 2–3 cm. We used “micro” class servos, but there are smaller ones available (“submicro” and “nano” class), which could be used to reduce the diameter even further (about 0.5–1 cm). The height of the flower (approx. 7 cm total in our system) is mostly limited by the length of the servo arm, which has to have enough vertical room to turn to the downward refill position. If the diameter is made smaller, the height can also be lower, as the servo arm is shorter in that case. The circuit boards of the flowers can be made more compact by fabricating them instead of using stripboard. The circuit board can actually be omitted altogether, by soldering components directly to the wires (the so‐called “dead bug” design) if a very small flower is desired. Also note that the flower does not have to be round as in our case, as the only factors limiting the geometry of the nectar container are the height and width in the servo arm's direction, which has to have enough space to allow the refill movements. Scaling up is more straightforward, simply using larger parts and if necessary, more powerful servos.

Both the firmware and control software are released under open‐source license and can be freely modified. The control software we provide here is flexible enough to be used in various experiments without modifications. However, modification of the source code allows the control program to be tailored to run any specific experiment.

One interesting possibility for modification is to change the behavior of the program from predetermined to dynamic. In the current version, the program runs according to preset parameters throughout the experiment. However, the rate of refill operations of independent flowers could be adjusted dynamically in response to learning of the animal, based on visitation data from sensors. For example, the refill rate of a specific (type of) flower could be increased or decreased when it is visited more often than other (types of) flowers.

## Conclusion

We have developed a complete, affordable, and automatic robotic flower (feeding) system for pollination and behavioral experiments. Our system not only handles flower refill operations but also data collection and storage, in a user‐friendly way. All software and hardware design are open source and freely modifiable.

The development of low‐cost open‐source microcontroller prototyping platforms in recent years made this system possible. We encourage other researchers to explore the possibilities of this approach.

## Conflict of Interest

None declared.

## Supporting information


**Figure S1.** An example of visitation data produced by our software, viewed in Microsoft Excel.

**Figure S2.** Circuit diagram of a single flower.

**Figure S3.** Circuit diagram of the connector cards of the control unit.

**Figure S4**. Use of the system.

**Figure S5**. Movement range of the servo arm: in refill position (left) and in normal feeding position (right).

**Figure S6**. Flower with the top cover removed.

**Figure S7**. Control unit.

**Figure S8**. Examples of individual flowers (with top cover in place) we used (Lämsä et al., unpublished).

**Appendix S1**. ZIP package file containing control software, its source code, firmware's sources and user instructions.
