# Supplementary figures and images for: A low‐cost, computer‐controlled robotic flower system for behavioral experiments
Source: Ecol Evol. 2016 Mar 16;6(8):2594–600. doi: 10.1002/ece3.2062 (PMC4798157; doi:10.1002/ece3.2062)

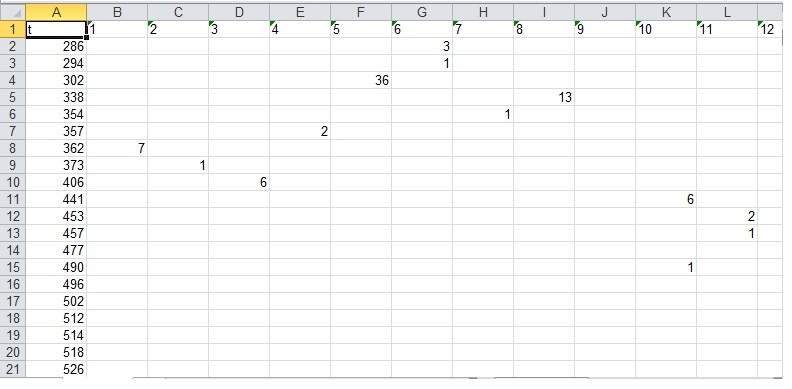

Supplement: Supplementary file 1 — Figure S1. An example of visitation data produced by our software, viewed in Microsoft Excel. [file ECE3-6-2594-s001.tif]

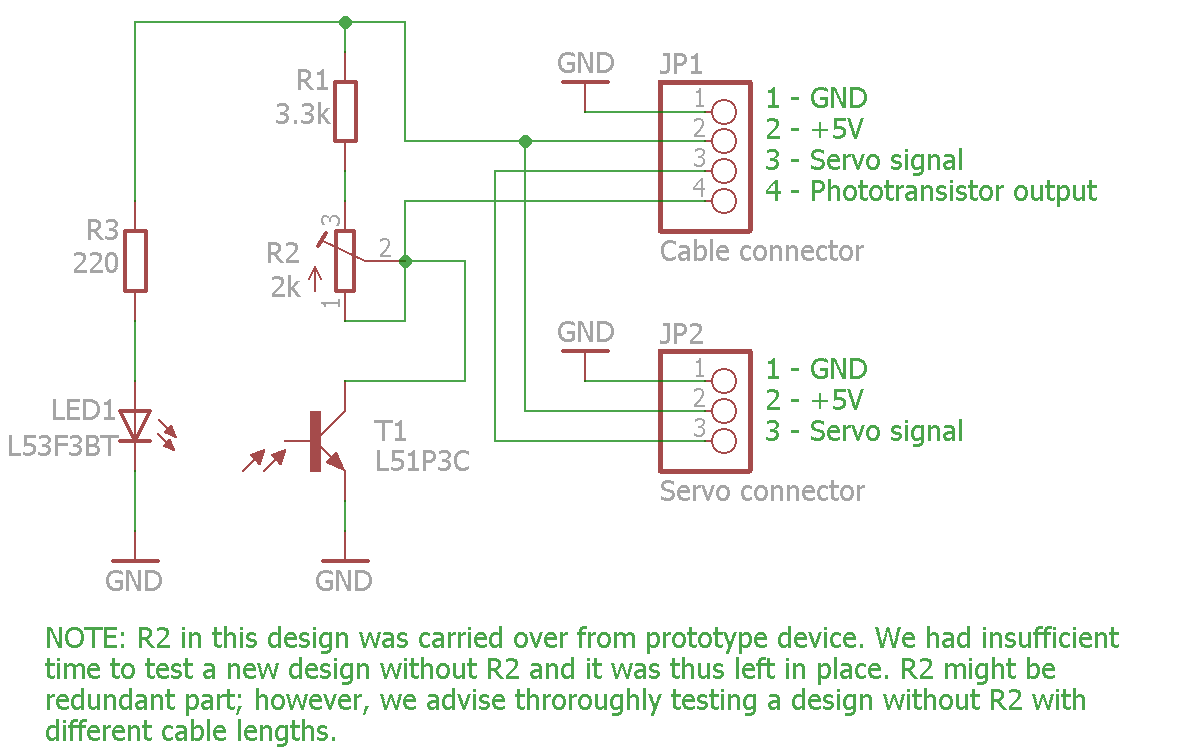

Supplement: Supplementary file 2 — Figure S2. Circuit diagram of a single flower. [file ECE3-6-2594-s002.tif]

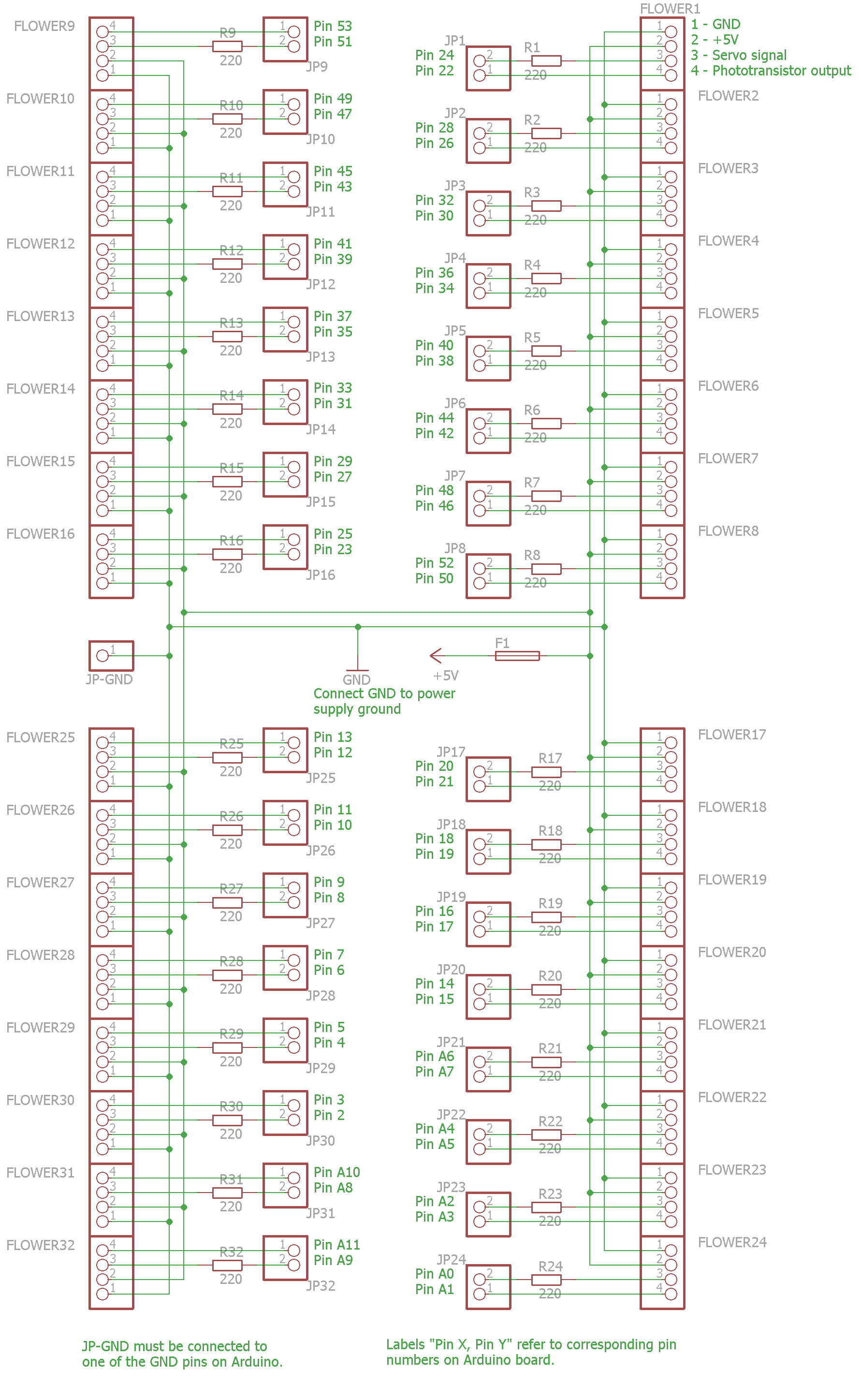

Supplement: Supplementary file 3 — Figure S3. Circuit diagram of the connector cards of the control unit. [file ECE3-6-2594-s003.tif]

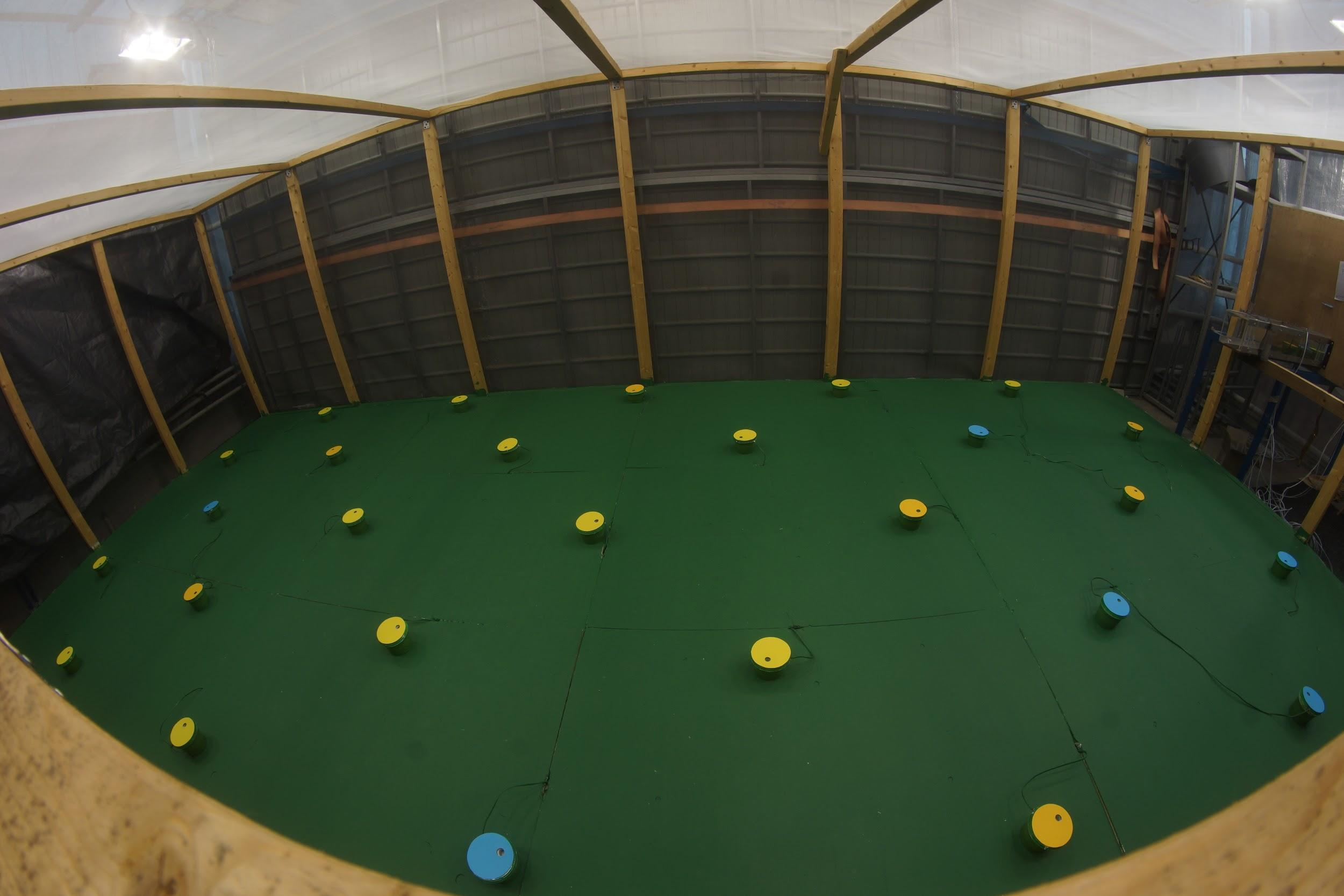

Supplement: Supplementary file 4 — Figure S4. Use of the system. [file ECE3-6-2594-s004.tif]

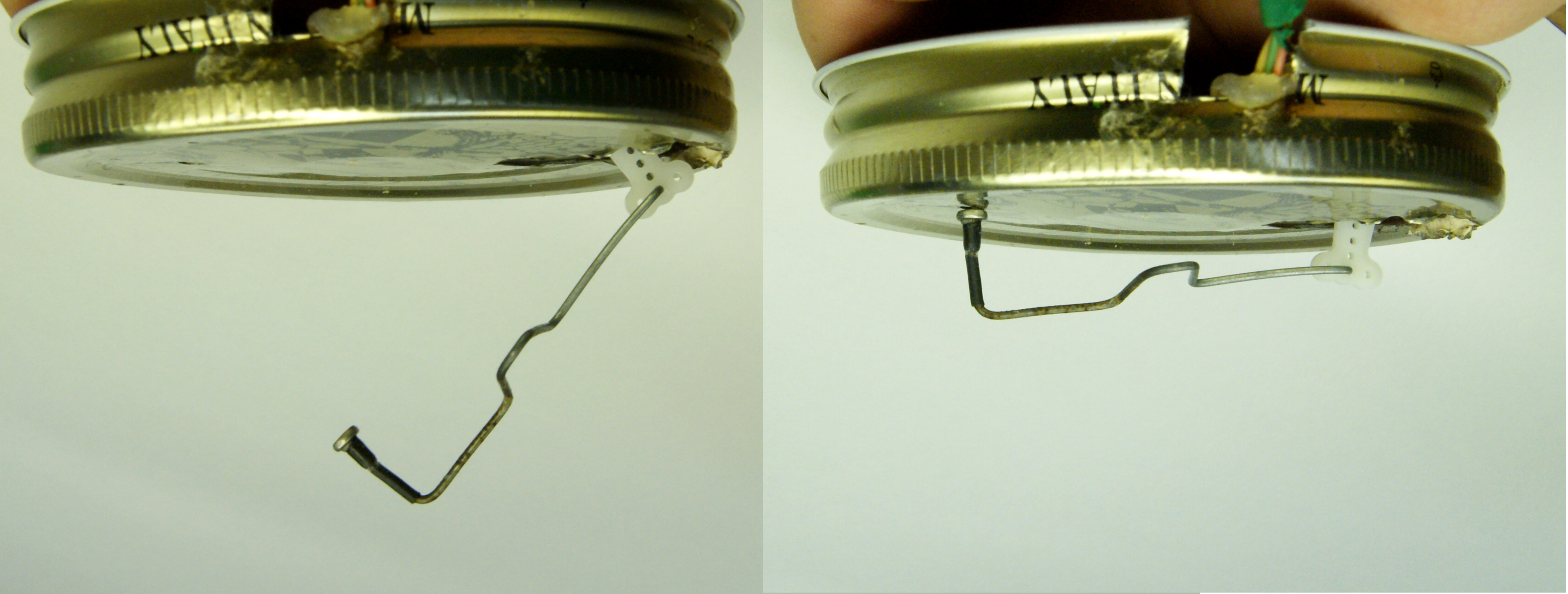

Supplement: Supplementary file 5 — Figure S5. Movement range of the servo arm: in refill position (left) and in normal feeding position (right). [file ECE3-6-2594-s005.tif]

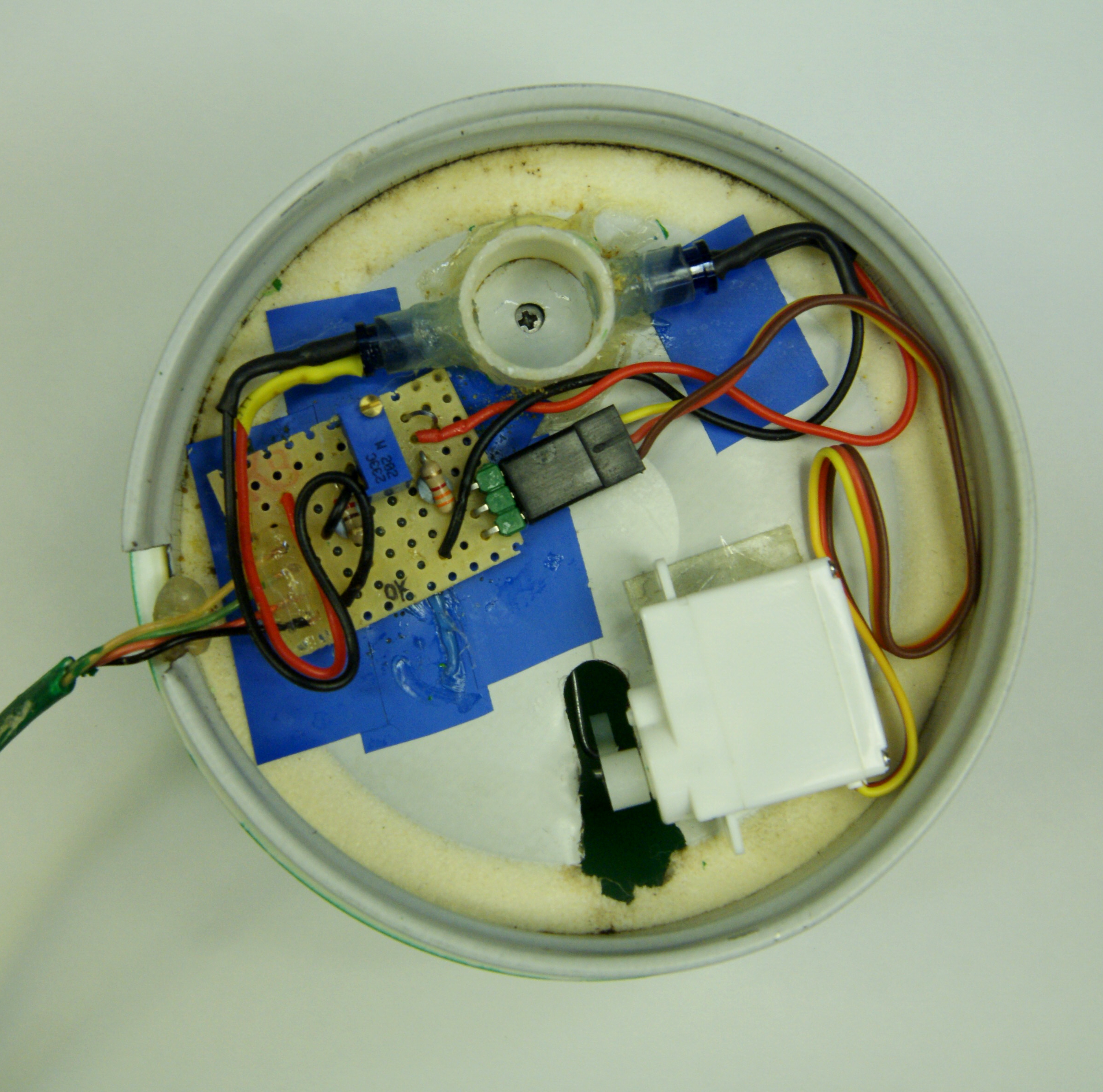

Supplement: Supplementary file 6 — Figure S6. Flower with the top cover removed. [file ECE3-6-2594-s006.tif]

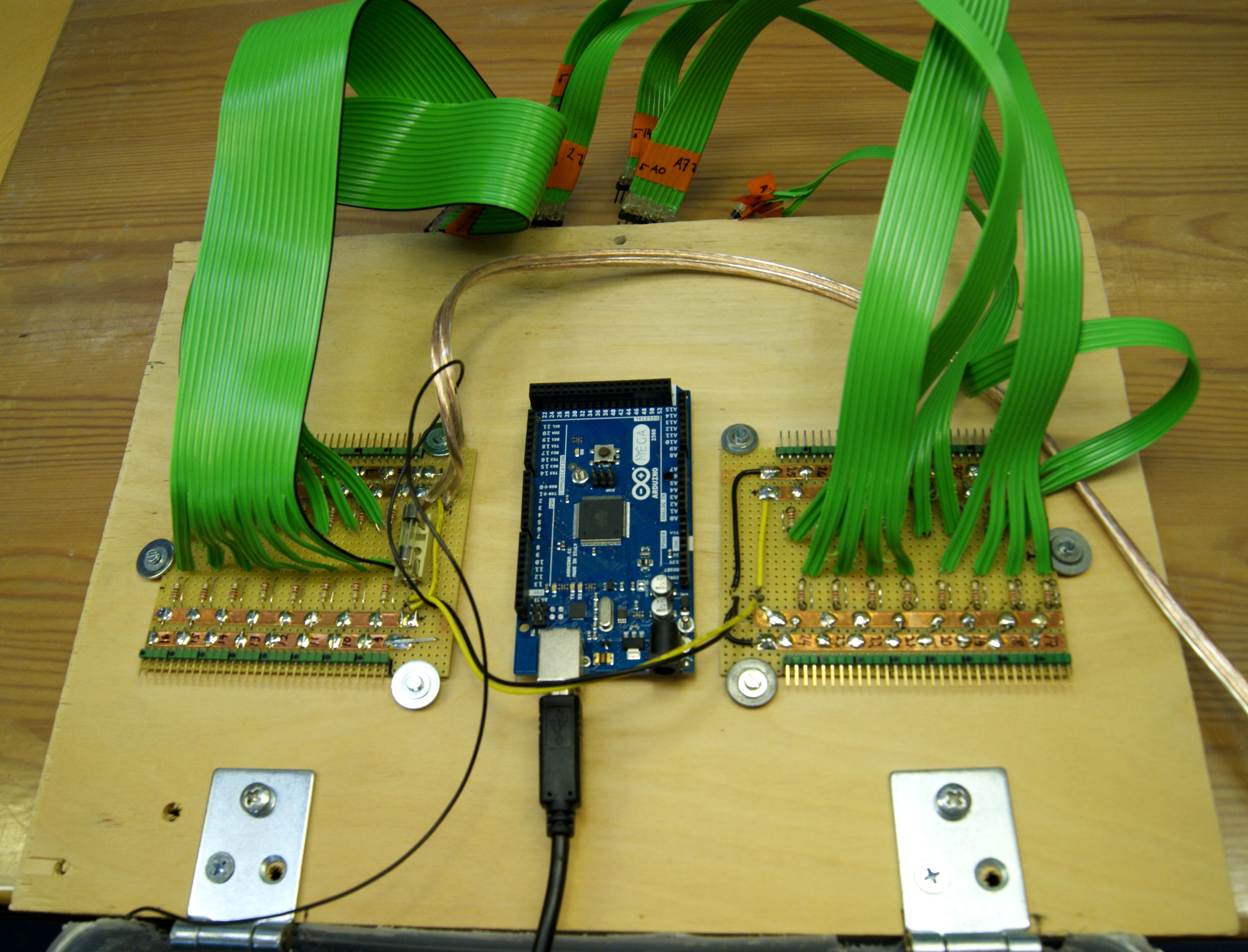

Supplement: Supplementary file 7 — Figure S7. Control unit. [file ECE3-6-2594-s007.tif]

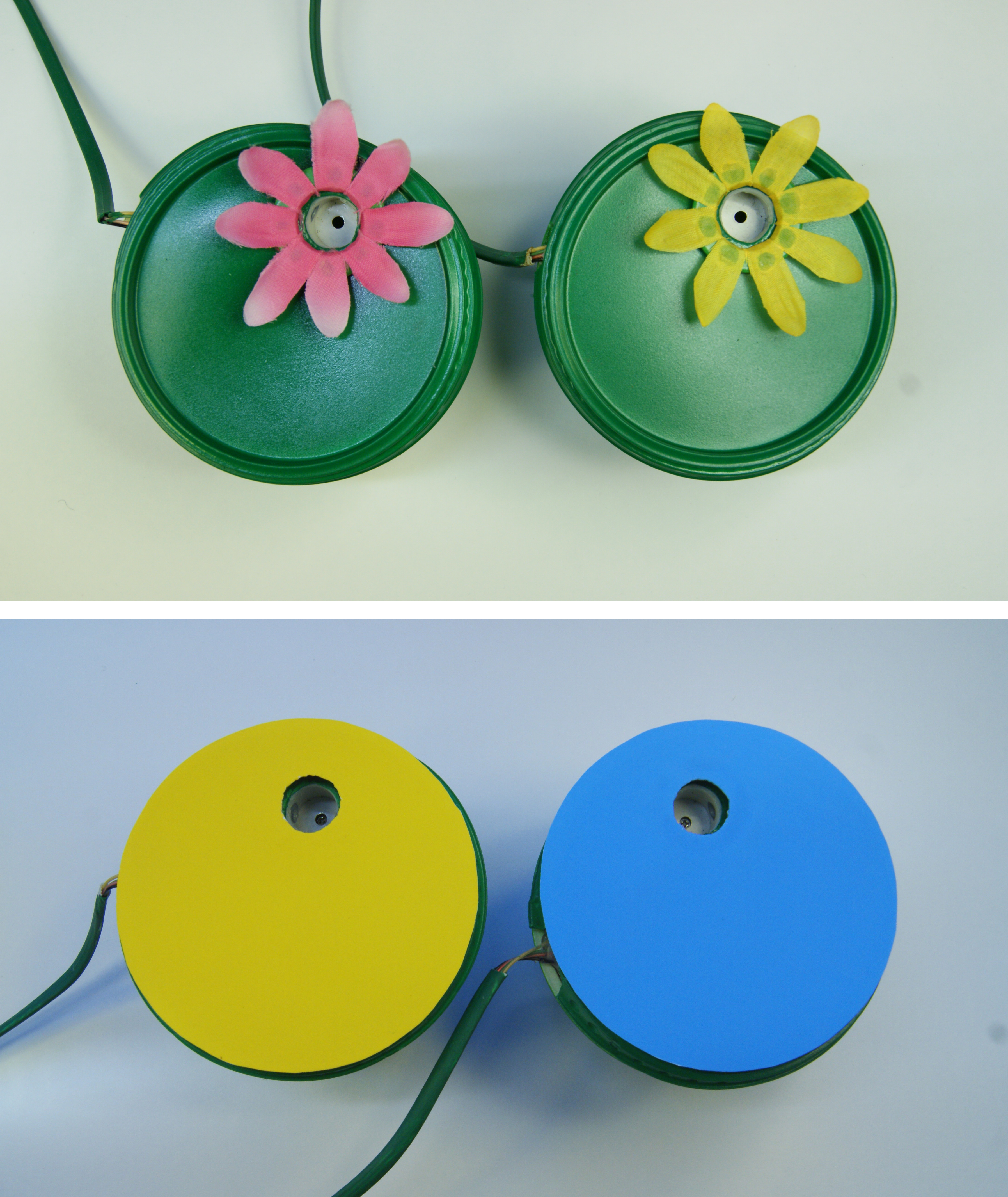

Supplement: Supplementary file 8 — Figure S8. Examples of individual flowers (with top cover in place) we used (Lämsä et al., unpublished). [file ECE3-6-2594-s008.tiff]
