# Supplementary material for: A low‐cost, computer‐controlled robotic flower system for behavioral experiments
Source: Ecol Evol. 2016 Mar 16;6(8):2594–600. doi: 10.1002/ece3.2062 (PMC4798157; doi:10.1002/ece3.2062)
Supplement: Supplementary file 9 — Appendix S1. ZIP package file containing control software, its source code, firmware's sources and user instructions. [file ECE3-6-2594-s009.zip › RoboticFlowerSystem/ControlApp/manual.pdf]

Control Application for Robotic Flower System – operating instructions

Installation

To run this application, following software needs to be installed:

- Java virtual machine. You can download its installation package from www.java.com.
- Arduino software, this can be found at www.arduino.cc.

Also, the firmware needs to be transferred to (programmed) the Arduino board. Please refer to separate instructions to do this. To install this application, extract the contents of this zip file to a directory where you have read/write permissions. For example, C:\ControlApp.

Starting the program

NOTE: Before starting, connect the control unit's USB cable to computer.

There are two executable batch files in the root directory, ControlApp32.cmd and ControlApp64.cmd. If the Java virtual machine installed on your computer is 32-bit version, run the program by double-clicking "ControlApp32.cmd" file. Otherwise (with 64-bit Java), use the "ControlApp64.cmd".

Connection panel

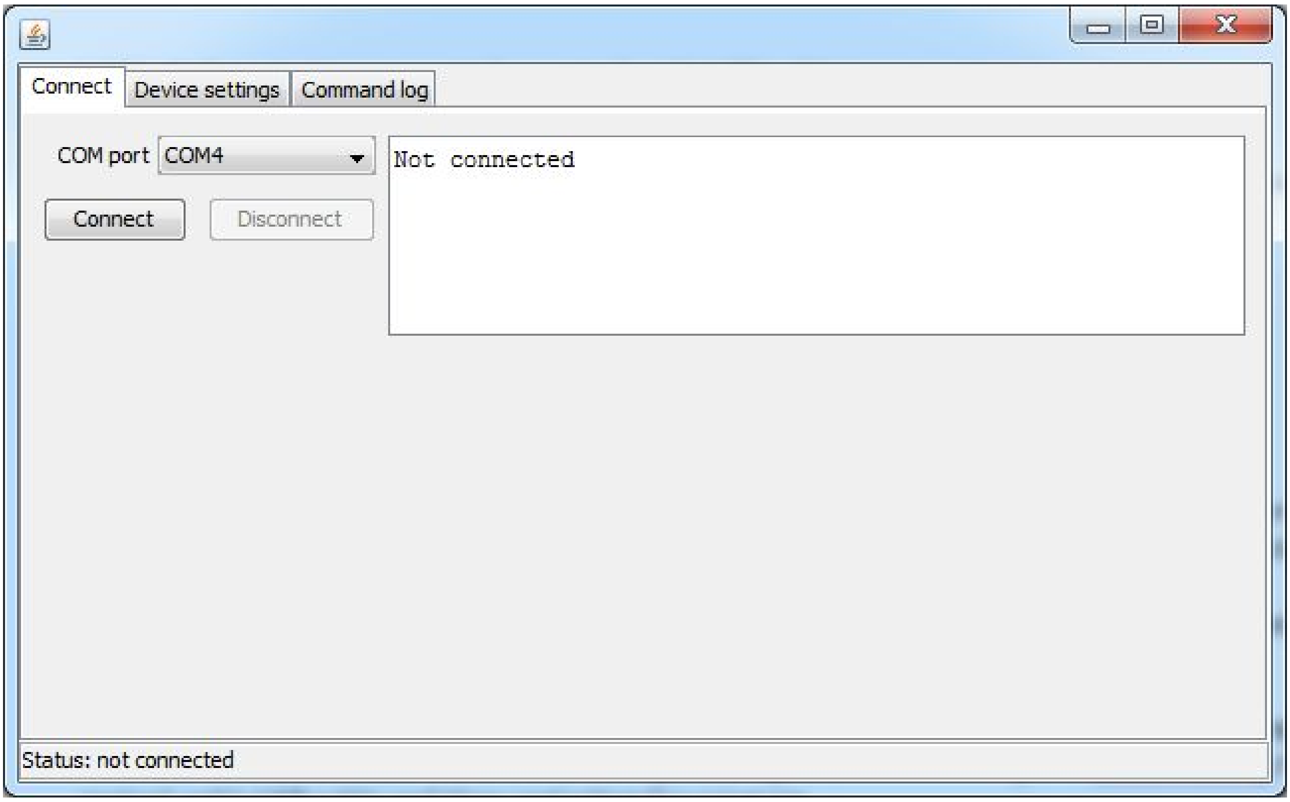

Figure 1. Connection panel.

The connection panel is the first view which is displayed to the user when program starts. It contains the components needed to make a connection the control unit. On the left side of the panel are drop-down list “COM port”, “Connect” and “Disconnect” buttons.

The drop-down list is used to select the communications port to which control unit is connected with an USB cable. This list might contain more than one item, depending on how many ports there are available on your computer. The “Connect” button is used to create the connection using the selected COM port, and “Disconnect” closes the existing connection.

On the right side of the panel there is a large text box. This is used to display information about the connection. When successful connection is made, text “Connected” is displayed here. Otherwise, if connection fails, an error message is displayed.

Instructions how to connect to the device are in section “Connecting to the control unit”.

Device settings

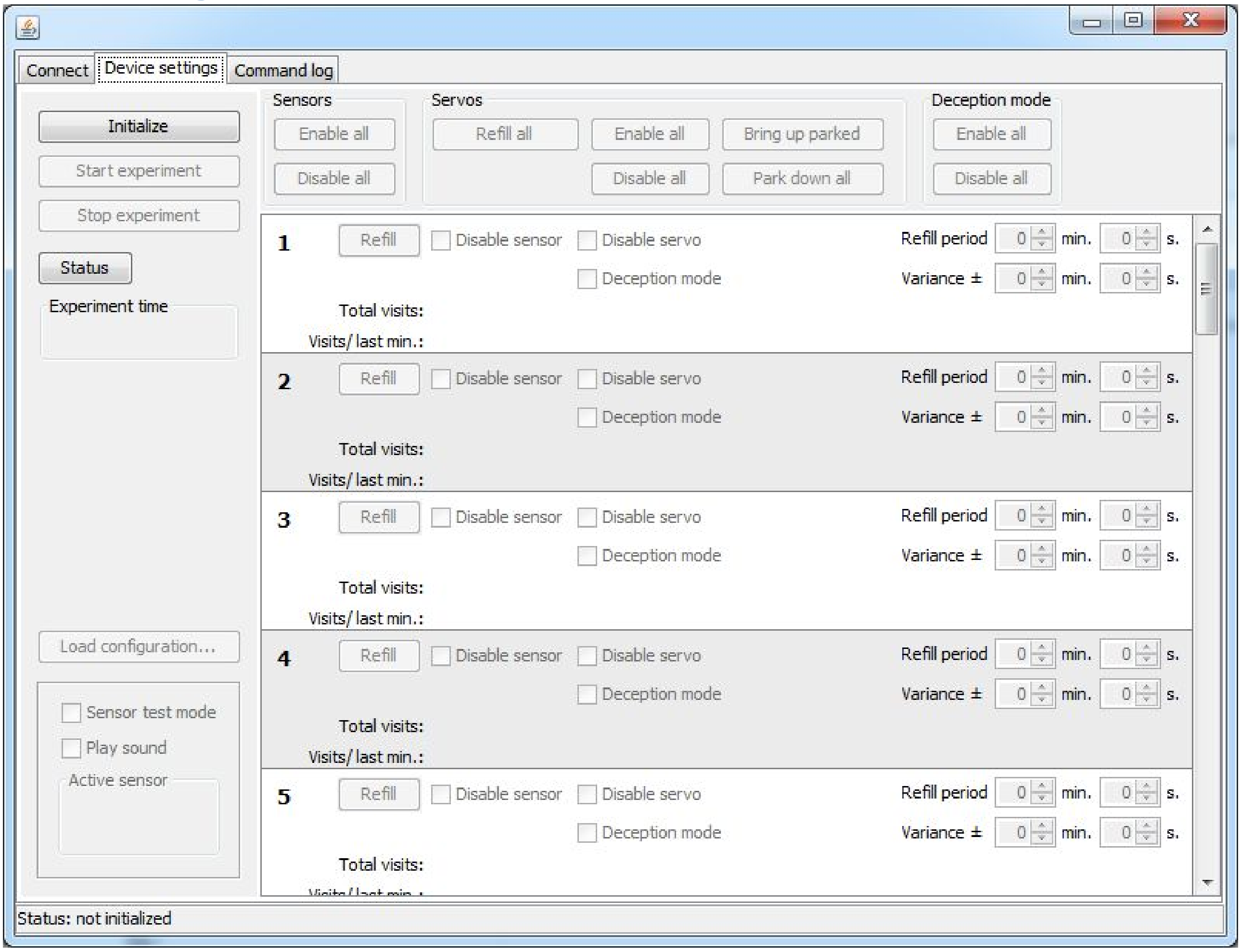

Figure 2. Overview of the Device settings panel

Device settings is the main user interface when using the software. On the left side panel are the main control buttons. On the topmost panel are shortcut buttons to control all the flowers simultaneously. The numbered items on the central part are the controls for the individual flowers.

Main controls

- Initialize: after the control unit has been powered on, it must reset all servos by driving them to normal position. This button orders the control unit to reset everything after start.
- Start experiment: this button is used to start the experiment after all flowers have been configured properly.
- Stop experiment: running experiment stops only after user decides to stop it. This button ends the experiment and converts collected data to Microsoft Excel file.
- Status. (this was only used in debugging the software and control unit)

- Load configuration: loads an Excel file containing predefined flower settings. See section “Configuration files”.
- Sensor test mode: when not running an experiment, this checkbox enables user to test sensors of individual flowers. When sensor is blocked manually, flower number is displayed below and length of the blocking period in seconds. Additionally, when “Play sound” is checked, notification sound is played (if computer has speakers connected) after each sensor test.

Top panel

- Enable/disable all (sensors). Enables or disables all sensors in all connected flowers. If sensor is disabled, it does not register any visits.
- Refill all (servos). This button commands all servos to perform refill operation.
- Enable/disable all (servos). Enables or disables all servos in all connected flowers. If a servo is disabled, it does not respond to refill commands.
- Park down all. This button commands all (non-disabled) servos to rotate the servo arm to downward position and stay there.
- Bring up parked. Commands all servos to return to normal position from “parked down” position.
- Enable/disable all (deception mode). Turns on or off “deception mode” for each flower. The servo arm is kept lower position, out of the animal’s reach when flower is in deception mode. The flowers respond normally to refill commands in this mode.

Central panel (flower controls)

This list contains individual settings for each flower connected to the system. Row number 1 represents flower #1, etc. For example, if flower #5’s servo is disabled (“Disable servo” checkbox is selected), it does not respond to refill commands, even when experiment is running. The refill button performs the refill operation for the specific flower.

Using this control panel, each flower’s refill rate can be individually configured. Period specifies how often refill operation is performed. For example, period 1 min. 15 s. makes control unit to refill flower first time after 1 minute and 15 seconds after start of experiment. After refill operation is completed (this takes about 14 seconds), timer is set again to 1 min. 15 s. and next refill is performed after that delay.

Refill operations can include randomness by setting variance parameters. For example, if flower’s refill period is configured to 1 min. 15 s., and variance is 15 s., then the refills happen in 1 minute and 1 min. 30 s. range. The random values are uniformly distributed. If variance is greater than refill period, a warning is displayed to user.

The flower list also displays simple statistics about each flowers: total visitation count and number of visitations during last full minute.

Command log

The command log panel displays the raw data what software sends to control unit and what it receives from it. All operations performed by the control unit are initiated by sending specific commands from the software. The commands are in text format, and are displayed in this panel along with time when the command was sent. Also, the responses and visitation data sent by the control unit are displayed on the same log.

Connecting to the control unit

Following steps are needed to make connection to the control unit.

1. Turn on computer
2. Connect the USB cable to the control unit and computer
3. Turn on power supply
4. Start the control software:
    a. If the Java installed on your system is 32-bit version, double-click the "controlApp32.cmd"
    b. If you have 64-bit Java installed, use the "controlApp64.cmd"
5. Once the software starts, from "Connect" panel, select appropriate COM port from the drop-down list and click "Connect". There may be more than one available port. If you don't know the port number, try each one.
6. If the connection was successful, text "Connected" appears on the large text box. Otherwise, an error message or "Device is not responding" is displayed. This might happen if wrong port was selected from the list, or there is some problem with the control unit itself.

To try another COM port, click "Disconnect" button, select another port and click "Connect" again. If none of the available ports work, try closing the program, disconnecting and re-connecting the control unit's USB cable and then restarting the program.

NOTE: On some computers, opening of the COM port takes long time for some reason (up to 9 seconds, and possibly even more). In these cases, the application's connection process may time out (maximum allowed delay is 12 seconds). If you get the message "Device is not responding", please wait for couple of seconds, then click "Command log" tab. If you see a row with green-colored text with message "S:0" or something similar (such as "S:2"), the device actually HAS responded correctly. In this case, you can start using the software normally.

Configuring the system

Before starting an experiment, each flower needs to be configured individually.

1. If the control unit has just been powered up, the device must be initialized. This ensures that all servos in each flower will be driven to normal position. If status bar on bottom of the program's window displays text "Status: not initialized", click button "Initialize" on device settings panel. The process takes few seconds to complete, and after it has been finished, status "initialized" is displayed on the status bar.
2. Once the device is initialized, flowers can be configured or operated manually. Additionally, sensor test mode can be enabled or disabled. The device is now in "ready" state.
3. To configure system for running an experiment, enter desired refill period values for each flower, and additionally enter variances. To disable sensor and/or servo, click corresponding check box on the flower list. Similarly, enable or disable deception mode for desired flowers. Alternatively, if you have predefined configuration file, use "Load configuration" button to load it (see section "Configuration files").
4. Once all flowers have been configured properly, the experiment can be started.

Running an experiment

1. After the steps in "Configuring the system" have been performed, start the experiment by clicking "Start experiment" button.
2. Controls in flower settings panel and top panel are disabled (to prevent user from altering the configuration during experiment). Simultaneously, a clock is started and displayed on main controls panel (on the left side of the screen). The clock measures the time elapsed since the start of the experiment.
3. Experiment continues to run according to set parameters. During this time, total visits to each flower (if sensor for the flower is enabled) are displayed as well as visits made during previous minute. When software commands a flower to refill, it is also displayed on the flower settings panel. During the experiment, visitation data from control unit is recorded to temporary file.
4. Experiment runs until user commands it to stop. To do this, click button "Stop experiment". Control unit goes back to "ready" state, and servos are returned back to either normal or deception mode position (depending how the flower was configured). Visitation data from temporary file is automatically converted to Microsoft Excel spreadsheet file, which is saved to user's home directory (the directory location varies among operating systems). The path to the Excel file is displayed in the status bar (bottom part of the screen): "Saved data to <path>", where <path> is the directory and file name. The file name consists of text "output-" with date in YYYYMMDD format and a running number, eg. "output-20151124-1.xlsx".
5. System is ready to be configured again (if needed) and new experiment can be started.

Configuration files

To speed up configuring the system for each experiment, flower settings (such as refill period) can be loaded from Excel file. When different flower configurations are used between experiments, loading predefined settings from a separate file speeds up configuring the device. This also minimizes the risk of makings errors in configuration. For example, when setting same refill period minutes field for each flower, user might enter incorrectly value "4" instead of intended "3" for some flower. This would cause the system to behave differently for this experiment and possibly would affect the results.

File "config.xlsx" in program's root directory contains blank sample configuration with instructions to how to enter values. To load the configuration file, click "Load configuration" button. A file selection dialog opens. Select the file you want to load, and click "Open". If the file is correctly formatted, flowers are configured according to its contents.
